# Addressing the cancer burden in LMICs: the potential of modulated electro-hyperthermia

**DOI:** 10.3389/fonc.2025.1590920

**Published:** 2025-12-16

**Authors:** Carrie Anne Minnaar, Mia Hugo, Prinitha Pillay, Thanushree Naidoo, Jeffrey Allen Kotzen, Duvern Ramiah

**Affiliations:** 1 Department of Radiation Sciences, University of the Witwatersrand, Johannesburg, South Africa; 2 Department of Radiation Oncology, Wits Donald Gordon Medical Centre, Johannesburg, South Africa; 3 Radiation Oncology, Dr Prinitha Pillay Inc., Johannesburg, South Africa

**Keywords:** low- and middle-income countries, HIV, cancer, cervical cancer, modulated electro-hyperthermia, resource-constrained settings, hyperthermia in oncology

## Abstract

Cancer in low- and middle-income countries (LMICs) is associated with late-stage diagnoses and poor survival, compounded by limited access to screening, diagnostic tools, and treatment. The high prevalence of HIV further complicates outcomes, especially in sub-Saharan Africa. Hyperthermia has emerged as a valuable adjunct to treatment in high-income countries, particularly for locally advanced cancers such as cervical and head and neck tumours, which are common in resource-constrained settings. Uptake of hyperthermia in LMICs has however been limited. A phase III randomised controlled trial in a South African public hospital demonstrated that modulated electro-hyperthermia (mEHT), a capacitively coupled heating technique, was feasible in this setting. The trial, the first to assess hyperthermia in Africa and to include HIV-positive participants, found that adding mEHT to chemoradiotherapy significantly improved 5-year disease-free survival (32% vs. 14%; HR:0.73; 95%CI:0.53-1.00; *p=0.049*); reduced recurrence, and improved quality of life without increasing toxicity. Benefits were observed equally in HIV-positive and HIV-negative participants. A cost-effectiveness analysis indicated an 82.2% probability of cost savings over three years, primarily due to fewer recurrences and residual disease and improved quality of life. Given its affordability, ease of integration, and lack of additional toxicity, mEHT represents a promising adjunct to chemoradiation in LMICs. Further replication in LMICs beyond sub-Saharan Africa is needed to confirm generalisability to diverse healthcare settings.

## Introduction

### Cancer care in low- and middle-income countries

Cancer continues to be a significant public health issue worldwide, but low- and middle-income countries (LMICs) bear the brunt of the burden. Low- and middle-income countries face unique challenges in cancer care, including limited access to screening, diagnostic facilities, and treatment options. As cancer detection rates and therapy successes increase in high-income countries, the World Health Organization (WHO) predicts that over the next decade more than 75% of cancer-related deaths will occur in LMICs. Almost 90% of countries in Sub-Saharan Africa are low-income or low-to-middle-income countries. The region is currently unprepared, with inadequate screening and detection methods, sub-optimal treatment, and limited palliative care capacity (1). The extreme socio-economic stress that most patients in these settings live with daily (2) impacts mental health which is an important prognostic factor for cancer (3). This review aims to evaluate the impact of modulated electro-hyperthermia (mEHT) on treatment outcomes and survival rates in cancer patients within resource-constrained settings, with particular focus on improving outcomes for HIV-positive individuals and in Sub-Saharan Africa.

#### Limited access to screening and diagnostic tools

One of the critical issues in LMICs is the limited awareness and availability of screening and diagnostic tools, resulting in a large proportion of cancer cases being diagnosed at advanced stages (4). The SURVCAN-3 study evaluated 10,500 individuals from 13 population-based cancer registries in 11 LMICs over 13 years. The study reported that 79% of cancer patients in sub-Saharan Africa are diagnosed in stage III and IV of disease, significantly impacting survival rates. Additionally, survival from cancers with a high burden and amenable to prevention was poor (4). This highlights the urgent need for early screening and detection. In LMICs, most diagnoses rely on clinical staging due to the lack of imaging facilities, which can lead to less accurate assessments of tumour size and spread (5, 6). This is especially problematic for cancers like cervical cancer, where accurate staging can guide treatment decisions (7). Early detection significantly improves treatment outcomes, but for many patients, this is not feasible due to financial, geographical, and resource constraints. Improving the management of advanced disease therefore becomes more important in these settings.

#### Suboptimal treatment access

Many LMICs lack the infrastructure for advanced radiation therapy. As of March 2020, only 28 of the 54 countries in Africa had access to radiation units, and only 21 countries had brachytherapy units (8). In some settings, low-energy machines like Cobalt-60 are still in use (8). The disparity in access to treatment extends beyond radiation to include drugs and specialised surgical interventions. Several international societies are developing tiered recommended treatment guidelines, with the most basic treatments as Tier 1 and the optimal, newest, and most effective combinations as Tier 4, to address the global variations in treatment access (9). With so few treatment facilities, patients are often forced to travel far to receive treatment, placing additional strain on the family socially, and financially (10). Although South Africa is classified as a middle- to upper- income country (11), there is a huge inequity in the access to healthcare, with only about 20% of the population having access to private healthcare (12). The rest of the population relies on a public healthcare system that is operating under severe constraints, with oncology facilities that are under-equipped and under-staffed to handle the high patient volumes. The same challenges are seen in public healthcare across Africa (13, 14).

#### Treatment compliance

Treatment compliance is impacted by factors such as transport limitations, work or family obligations, and treatment facilities which are not functioning (15, 16). Limited resources, too few healthcare providers, and communication barriers also negatively impacts on patient management and the patients’ understanding of the need to comply to treatment (17–20). One study in a public healthcare facility in South Africa showed that patients receiving brachytherapy experienced fear, pain, and humiliation associated with the treatment, bringing to light the need for the optimisation of pain management and patient care (21) to improve compliance and reduce the risk of patients electing not to have treatment. HIV infection, which is high in many developing countries, is also associated with a higher risk of incomplete treatment schedules and residual tumours (22, 23).

#### Human immunodeficiency virus and cancer in Sub-Saharan Africa

The relationship between HIV and cancer is well-documented, particularly in LMICs, where the burden of both HIV and cancer is high. According to the WHO, the HIV/AIDS epidemic continues to be a public health threat, with an estimated 39.9 million people living with the virus globally at the end of 2023, and 65% (+-25.935 million) residing in the African region (24). The immunosuppression caused by HIV allows for the unchecked proliferation of oncogenic viruses like human papillomavirus (HPV), Epstein-Barr virus (EBV), and the Kaposi’s sarcoma-associated herpesvirus (KSHV) (25, 26). People living with HIV (PLWH) are therefore at a higher risk of developing certain cancers, particularly those associated with viral infections like Kaposi’s sarcoma, non-Hodgkin lymphoma, and cervical cancer.

Recent studies have shown that while antiretroviral therapy (ART) has significantly reduced the incidence of some Acquired Immuno-Deficiency Syndrome (AIDS)-defining cancers, such as Kaposi’s sarcoma, PLWH still have a higher incidence of certain cancers compared to the general population. This includes non-AIDS-defining cancers such as lung, liver, and anal cancers (27). In fact, there appears to be an increase in non-AIDS defining cancers in PLWH (28). After evaluating cancer incidence in 5.2 million people with HIV in South Africa, Ruffieux et al. found that in young PLWH, infection-related cancers were dominant, whereas most cancer diagnoses in PLWH above the age of 54 years were infection-unrelated (29). The persistence of cancer risk despite ART highlights the need for comprehensive cancer prevention and treatment strategies in this population.

People living with HIV are at risk of lower cancer-related survival (30, 31), and outcomes compared with the general population, although this does vary based on cancer type, treatment availability and ART administration. For example, HIV-infection was associated with a higher hazard ration of death (HR:1.50; 95%CI:1.22–1.85; p<0.001) and worse disease-free survival (DSF) (OR:2.63; 95%CI:1.71–4.03; p < 0.001) in breast cancer patients in South Africa (32). In a separate study in South Africa, women living with HIV with stage IV hormone receptor-negative breast cancer had shorter overall survival (OS), compared to HIV-uninfected women (1-year OS: 27.1% vs. 48.8%, p=0.003; HR 1.94; 95%CI:1.27–2.94; p=0.002), however this difference was not observed for the sub group of women with hormone receptor-positive breast cancer (33). Overall, the survival differences between PLWH and cancer in the literature range from no difference (33–35), to significant differences (32, 33), such as the difference in median OS seen for PLWH with head and neck cancer of 34m [18-84] vs. 94m [86-103] for HIV-uninfected head and neck cancer patients (36).

Treating HIV-associated cancers requires a multidisciplinary team to manage concurrent opportunistic infections, potential drug-drug interactions, comorbidities and the co-occurrence of more than one cancer in the same patient. Many factors may lead PLWH to receive inappropriate dose adjustments, exclusion from emerging therapies and clinical trials, or no cancer therapy at all (37). HIV-positive patients with cancer are also more likely to experience complications during treatment. A clinical cohort study of 196 adults with HIV and cancer found that chemotherapy and/or radiotherapy resulted in an estimated decline in CD4 count of 203 cells/μL shortly after treatment, although no increase in HIV RNA levels was reported. Every decline in CD4 count of 100 cells/μL was associated with a 27% increase in mortality (38). Many early ARTs had toxic effects that overlapped with cancer therapies. Furthermore, an important class of ART, protease inhibitors, has clinically significant CYP3A4-mediated drug-drug interactions that may increase the toxic effects of many common antineoplastic agents (39). Additionally, toxicities from radiation may be higher in HIV-positive patients (40), emphasising the importance of radiosensitisers that do not alter the toxicity profile of radiation therapy.

### Hyperthermia

Hyperthermia is the application of heat to sensitise tumours to chemotherapy or radiotherapy. After reviewing randomised controlled trials on radiotherapy with/without hyperthermia Datta et al. calculated that the Biologically Effective Dose (BED) could be increased from 47.2Gy to 89.2Gy for locally recurrent breast cancer, from 79.1Gy to 141.9Gy for locally advanced head and neck cancer, and from 59.9Gy to 84.2Gy for locally advanced cervical cancer (LACC), and in all cases the alpha/beta ratios appeared to be lowered from 10 to between 2 and 4. The radiodensitising effect of hyperthermia therefore has potential to lower the alpha/beta ratios of the tumours and increase the BED (41). There are numerous devices used to heat tumours, with variations in energy delivery methods and dose control. Some technologies integrate MR-guided thermal mapping, requiring sophisticated settings and specialised staff. Most trials on hyperthermia have been conducted in Europe, Asia and America and the benefits of the addition of hyperthermia have been demonstrated in tumours in the head and neck region (42, 43), cervical cancer (44–49), high risk soft tissue sarcomas (50), recurrent breast cancers (51, 52), brain tumours (53), local recurrences in previously irradiated fields (54–60), bone metastases (61), and pancreatic tumours (62). In meta-analyses of randomised controlled trials, Datta et al. showed that the addition of hyperthermia resulted in an average increase in response rates by 22% in head and neck tumours (43), cervical tumours (63) and in recurrent breast tumours (51).

Hyperthermia for the management of cervical cancer has been extensively studied in upper-income countries (44, 45, 47, 48, 64–66). In their article on the use of hyperthermia as an adjunct for the treatment of LACC in the Netherlands, Van der Zee et al. demonstrated that the addition of hyperthermia to radiotherapy resulted in a maximum discounted-cost-per-life-year gained of 4000 Euros, with significant improvement in survival in the hyperthermia group at the three years (27% vs. 51%; *p=0.009*) and at twelve years (37% vs. 56%; *p = 0.01*) (45, 49).

### Modulated electro-hyperthermia

In a market research report, high initial investment costs for equipment and infrastructure are listed as significant barriers for the uptake of magnetic resonance hyperthermia technologies, especially in developing countries (67). This, along with the maintenance and skilled staff required, are likely the main reasons why hyperthermia uptake in LMICs has been low, despite the treatment having been around for decades. This is a general problem seen in healthcare in Africa where the initial investments, skilled personal required, infrastructure, and downtime due to maintenance challenges, impact the roll-out of technologies such as radiotherapy and imaging devices (15, 68, 69).

Modulated electro-hyperthermia is a novel heating technique that uses 13.56MHz radiofrequency waves transmitted between two capacitively-coupled electrodes placed on either side of the patient. Amplitude modulation is applied to the radio waves to enhance the effects of the treatment on the tumour and impedance matching allows for the focussed and selective energy deposition in malignant tissues. The technology allows for non-invasive treatments that are easily delivered without the need for sophisticated imaging or specialised facilities or staff. Motivated by the cost-benefit seen in the trial by van der Zee et al. (45), a trial was designed to test the effects of the addition of modulated electro-hyperthermia in a public hospital in South Africa. This was the first trial on hyperthermia in Africa, in a resource-constrained setting, and that included HIV-positive participants. The trial enrolled 210 women, HIV-negative and -positive, with LACC and the participants were randomised to receive either chemoradiotherapy alone or in combination with mEHT. Local disease control was evaluated, and participants were followed up for five years post-treatment. The results of the trial are summarised in **Tables 1** and **2**.

**Table 1:** Results from the South African LACC trial.

Definition (per source)	mEHT + CRT	CRT alone	Effect size	p-value	Ref
CMR of all in-field disease at 6m (PET/CT)	45.5% (40/88)	24.1% (20/83)	Chi Squared	*0.003*	(7)
DFS at 2y (KM)	36.4% (36/99)	13.7% (14/102)	HR:0.67 (95% CI 0.48–0.93)	*0.017*	(70)
DFS at 3y (KM)	35.4% (35/99)	13.7% (14/102)	HR:0.70 (95% CI: 0.51-0.97)	*0.035*	(70)
OS at 3y (KM)	44.0% (44/100)	33.7% (34/101)	HR:0.72 (95% CI:0.51-1.03)	0.074	(70)
CMR of all disease incl. extra-pelvic disease (PET/CT) at 6m	24.1% (13/54)	5.6% (3/54)	Fisher’s exact	*0.007*	(71)
DFS at 5y ^+^	32% (32/99)	14% (14/102)	HR:0.73 (95% CI:0.53-1.00)	*0.049*	(72)

CRT, Chemoradiotherapy; CMR, Complete metabolic response; DFS, Disease-free survival; mEHT, Modulated electro-hyperthermia; OS, Overall survival; PET/CT, Positron emission tomography/computed tomography. **HIV subgroups:** Improvements in LDC and DFS were observed in both HIV-positive and HIV-negative participants; CMR of extra-pelvic disease was seen equally in HIV-positive and -negative participants and was not associated with the number of cisplatin doses. **^+^**Five-year DFS derived from a peer-reviewed conference abstract (ESTRO 2024). Full manuscript pending.

**Table 2:** Quality of life and toxicity results from the South African LACC trial.

Outcome	Measurement	Finding	HIV-subgroup
Early RT toxicity (73)	On-treatment AEs and CTCAE v4 at 6m post-treatment	No significant differences between treatment groups	Similar patterns in HIV+ vs HIV–
Late RT toxicity (72)	Assessed up until 5y post-treatment	No significant differences between treatment groups	Similar patterns in HIV+ vs HIV–
QoL (70)	Assessed at regular intervals for 2y post-treatment using the EORTC QLQ-C30/Cx24	Significant improvement in 10/11 categories in the mEHT group at 2y post-treatment vs. a significant improvement in 3/10 in the control group	Benefits seen regardless of HIV status

AEs, adverse events; HIV, Human immunodeficiency virus; mEHT, Modulated electro-hyperthermia; RT, Radiation therapy. **HIV subgroups:** there were no significant differences in toxicity or quality of life reported based on the HIV subgroups.

#### Affordability and feasibility

A cost-effectiveness analysis used a Markov model to evaluate data from the South African LACC trial. The model showed an 82.2% probability that the addition of the mEHT would result in a saving for the public healthcare facility (70). The cost-saving is a result of fewer participants with recurrences and residual disease post-treatment requiring second-line treatment. The technology’s affordability, non-invasiveness, safety and ease of integration into existing treatment protocols, mean that it can be deployed without requiring significant additional infrastructure or training in a resource-constrained setting.

#### Local disease control and survival benefits

The addition of mEHT to the standard treatment more than doubled the five-year DFS rates in the South African LACC trial. At five years, the DFS rates were 32% vs. 14% (OR 3.0, *p=0.002*), corresponding to a hazard ratio of 0.73 (95% CI 0.53–1.00; *p=0.049*). This resulted in a significant reduction in recurrences, leading to improved outcomes in a vulnerable population with limited access to optimal care (70). This improvement is critical in settings where late-stage diagnosis and limited treatment options contribute to high mortality rates. Moreover, mEHT’s ability to enhance tumour control can reduce the need for extensive follow-up treatments, which are often challenging for patients in LMICs due to travel and financial constraints.

Notably, 54 women in each treatment group had extra-pelvic metastatic disease and 24% of the women in the mEHT group achieved complete metabolic resolution of all disease at six months post-treatment, despite their advanced stage, HIV-status and poor prognosis (71). All but two of the women remained disease-free five years post-treatment and the two who passed away died from non-cancer related causes (72). It has long been suggested that hyperthermia has immune-modulating effects (74). This systemic response to the treatment suggests that mEHT may be able to potentiate an immune response in participants with stage IVB disease (71). The abscopal response is an immune-mediated response to radiation which results in a response in lesions outside of the primary treatment field. Interestingly, in the trial the abscopal response was also seen equally in HIV-positive participants, despite their compromised immune system.

#### Quality of life

Participants who received mEHT in the South African LACC trial had significantly higher quality of life scores two years post-treatment than those who did not receive mEHT. Women who received mEHT returned to work earlier and reported higher social, physical, and emotional functioning (70). This has potential to ease the strain on the healthcare system, the socioeconomic system, and the community.

#### Vulnerable and HIV-positive population

Around 50% of the participants in the cervical cancer trial in South Africa were HIV-positive. The improved outcomes in the mEHT group were seen equally in the HIV positive and negative participants and there was no difference in the toxicity profiles between the HIV-positive and -negative participants in the mEHT group (7, 70, 73).

## Discussion

Cervical cancer is a significant health challenge, particularly in LMICs, where 85% of cases occur. In 2022, an estimated 94% of the 350–000 deaths caused by cervical cancer occurred in LMICs (75). Factors such as high HIV prevalence, limited HPV vaccination coverage, and inadequate screening contribute to the high incidence and mortality rates of cervical cancer in these settings. Limited access to diagnostic tools and inadequate treatment facilities further exacerbates this burden. As mEHT augments the biological effectiveness of chemoradiation without reducing prescribed RT dose, or increasing toxicity, the addition of mEHT to standard chemoradiation presents a viable, cost-effective intervention for cervical cancer (70). This has potential to significantly improve treatment outcomes and reduce the strain on overburdened healthcare systems in Sub-Saharan Africa.

In the South African study, only basic 2D planning was available with older linear accelerators. Due to resource constraints, participants could only receive a maximum of two doses of cisplatin, and higher doses were given to compensate. However, the higher doses frequently resulted in haematological toxicity, and as a result not all participants could receive the second dose, while some could not receive any cisplatin. Despite these limitations, the addition of mEHT significantly improved the efficacy of treatments by sensitising tumours to radiation, without increasing toxicity to healthy tissues (72). While these features may be seen as limitations in studies conducted in upper-income countries, they reflect the reality of cancer-care in LMICs. Demonstrating improved outcomes under such constraints strengthens the validity of the results for LMICs, particularly those in Sub-Saharan Africa. Modulated electro-hyperthermia measures the energy deposited in the tumour through real-time impedance feedback, ensuring safe and effective treatment without invasive thermometry. This is a practical advantage that may help reduce barriers to adoption of HT in low-resource contexts. The primary limitation of the study in this context is that it was single centre; multicentre replication across diverse LMIC settings is needed to confirm generalisability.

The outcomes seen in the South African LACC trial are in line with the trials which investigated hyperthermia for the management of LACC in high-income countries such as the Dutch Deep Hyperthermia trial (45, 49), and the work by Franckena et al. (64), Harima et al. (53, 54, 65, 66), and Datta et al. (44, 76), suggesting that hyperthermia, which is generally seen as a treatment for more sophisticated settings, may also play a beneficial role in resource-constrained settings and should not be overlooked for these settings. However, generalisability to other LMICs outside of Sub-Saharan African with different cancer burdens, HIV prevalence, and health system constraints still requires some investigation.

In their 2022 paper, Datta et al. from India and Switzerland, argued that hyperthermia could be a transformative adjunct to treatment in LMICs owing to the affordability and potential to improve outcomes (77). According to Datta et al., locally advanced head and neck cancers, LACC, and locally recurrent breast cancers account for one third of all cancers seen in LIMCs. All of these cancer types have level 1 evidence in the form of meta-analyses of randomised controlled trials showing the benefit of the addition of hyperthermia. The meta-analyses showed that hyperthermia improves local tumour control by 22.1% (p < 0.001); 22.3% (p < 0.001); and 25.5% (p < 0.001), for the management of LACC; locally recurrent breast cancer; and locally advanced head and neck cancers respectively (41).

While the South African LACC trial results align with Datta et al.’s argument for the inclusion of hyperthermia in LMICs, there are practical barriers to implementation which remain a challenge. Device acquisition and maintenance require reliable supply chains and technical support, which may be a major limiting factor. Although specialised staff and facilities are not required, staff training and support is still necessary as are basic facility requirements such as stable electricity. Additionally, health systems in LMICs face competing priorities, and even if mEHT is cost-effective in the long term, initial investment in equipment and training competes with urgent needs such as vaccines and essential medicines. Addressing these barriers will require partnerships between governments, international organisations, and manufacturers, as well as phased roll-out in high-burden centres. In 2015, the WHO recognised mEHT as an innovative technology for low resource settings, prompting further investigations into its use in these settings (78).

Lastly, regulatory and financing considerations also differ by setting: in the public sector, where patients are not billed individually, integration depends on recognition within hospital budgets and national cancer control plans; whereas in mixed health systems, insurance reimbursement may be challenge. Addressing financing pathways will therefore also be a prerequisite for the wider implementation of mEHT in LMICs. More research into the development of strategies for scaling up mEHT, including maintenance models, training programs, and integration into tiered treatment guidelines, is warranted. If effectively implemented, mEHT could become a valuable adjunct in addressing the disparity in cancer outcomes between LMICs and high-income countries, particularly for vulnerable populations such as people living with HIV.

## Conclusion

As LMICs continue to struggle with the growing cancer burden, mEHT presents a promising feasible and accessible adjunct to treatment that may reduce the burden of cancer on healthcare systems in resource-constrained settings in Sub-Saharan Africa. While numerous randomised trials from high-income countries have already demonstrated the benefits of hyperthermia, further replication in LMICs beyond sub-Saharan Africa would confirm generalisability to diverse healthcare settings.
